# Gene Expression Profiling Specifies Chemokine, Mitochondrial and Lipid Metabolism Signatures in Leprosy

**DOI:** 10.1371/journal.pone.0064748

**Published:** 2013-06-14

**Authors:** Luana Tatiana Albuquerque Guerreiro, Anna Beatriz Robottom-Ferreira, Marcelo Ribeiro-Alves, Thiago Gomes Toledo-Pinto, Tiana Rosa Brito, Patrícia Sammarco Rosa, Felipe Galvan Sandoval, Márcia Rodrigues Jardim, Sérgio Gomes Antunes, Edward J. Shannon, Euzenir Nunes Sarno, Maria Cristina Vidal Pessolani, Diana Lynn Williams, Milton Ozório Moraes

**Affiliations:** 1 Laboratório de Hanseníase, Instituto Oswaldo Cruz, Fundação Oswaldo Cruz, FIOCRUZ-RJ, Rio de Janeiro, Brazil; 2 Laboratório de Pesquisa em Farmacogenética, Instituto de Pesquisa Clínica Evandro Chagas (IPEC), FIOCRUZ-RJ, Rio de Janeiro, Brazil; 3 Laboratório de Microbiologia Celular, FIOCRUZ-RJ, Rio de Janeiro, Brazil; 4 Instituto Lauro de Souza Lima, Bauru, São Paulo, Brazil; 5 Health Resources and Services Administration (HRSA), Bureau of Primary Health Care (BPHC), Division of National Hansen's Disease Programs, Laboratory Research Branch at the School of Veterinary Medicine, Louisiana State University, Baton Rouge, Louisiana, United States of America; University College Dublin, Ireland

## Abstract

Herein, we performed microarray experiments in Schwann cells infected with live *M. leprae* and identified novel differentially expressed genes (DEG) in *M. leprae* infected cells. Also, we selected candidate genes associated or implicated with leprosy in genetic studies and biological experiments. Forty-seven genes were selected for validation in two independent types of samples by multiplex qPCR. First, an *in vitro* model using THP-1 cells was infected with live *Mycobacterium leprae* and *M. bovis* bacillus Calmette-Guérin (BCG). In a second situation, mRNA obtained from nerve biopsies from patients with leprosy or other peripheral neuropathies was tested. We detected DEGs that discriminate *M. bovis* BCG from *M. leprae* infection. Specific signatures of susceptible responses after *M. leprae* infection when compared to BCG lead to repression of genes, including *CCL2*, *CCL3*, *IL8* and *SOD2*. The same 47-gene set was screened in nerve biopsies, which corroborated the down-regulation of *CCL2* and *CCL3* in leprosy, but also evidenced the down-regulation of genes involved in mitochondrial metabolism, and the up-regulation of genes involved in lipid metabolism and ubiquitination. Finally, a gene expression signature from DEG was identified in patients confirmed of having leprosy. A classification tree was able to ascertain 80% of the cases as leprosy or non-leprous peripheral neuropathy based on the expression of only *LDLR* and *CCL4*. A general immune and mitochondrial hypo-responsive state occurs in response to *M. leprae* infection. Also, the most important genes and pathways have been highlighted providing new tools for early diagnosis and treatment of leprosy.

## Introduction

Gene expression signature differences between bacille Calmette Guerin (BCG) vaccine strains and virulent mycobacteria, such as *Mycobacterium leprae* and *M. tuberculosis*, may help to understand the mechanisms triggered by virulent strains in order to escape microbicidal pathways and regulate the cellular microenvironment into a safe niche for replication. Indeed, previous studies have indicated that *M. leprae* is a deactivator of monocytes, macrophages and dendritic cells *in vitro* [1], [2], [3], suggesting that *M. leprae* plays an active role in shaping the cellular response towards a phagocytic and anti-microbicidal program [4] that, consequently, modulates the release of cytokines. The elucidation of the cytokine activation pathways from the early interactions between *M. leprae* and response immune cells should facilitate a better understanding concerning the progression to disease.

Pathogenesis routes in leprosy initiate with the recognition of pathogen-associated molecular patterns (PAMPs) from *M. leprae* by pattern recognition receptors (PRRs) and mycobacterial uptake (*TLR*, *NOD2* and *MRC1*). These genes have been associated with leprosy outcome in genetic epidemiological studies [5], [6], [7], [8], [9], [10]. Further, there is a cytokine production trigger through NF-kB and vitamin D receptor pathways, which culminate in the production of proteins directly involved in microbicidal activity. The ability of *M. leprae* to down-modulate these pathways provides a specific niche for its replication, survival, and successful infection [11], [4].

Also, experimental data, genomic scans and genome wide association studies (GWAS) pinpointed several genes associated with leprosy and refined main pathways associated with disease [10]. Zhang et al. (2009) identified by GWAS six genes associated with leprosy resistance/susceptibility, some of which have been replicated [6], [12], [13] including *NOD2*. Several genes involved in the pathogenesis of leprosy have been observed, such as *PARK2*/*PACRG*, *LRRK2* and *TNFSF15*, participating in the regulation of host-cell apoptosis [12], [14], and also genes that participate in the formation and maintenance of granulomas, such as *TNF*, *LTA* and *IFNG* [15], [16], [17], [18]. In case-control studies, SNPs in the *IL10* gene (−819 C>T) [19] and *IFNG* gene (+874 T>A) [16] were reported to be associated with susceptibility and protection in leprosy, respectively.

Finally, several genes clustered in the 17q11–q21 region, such as chemokines *CCL2*, *CCL3*, *CCL4*, *CCL5* and *CCL7*, were also linked to leprosy [20], [21]. Also, another gene recently identified as associated to leprosy is *LTA4H* (leukotriene A4 hydrolase), in which SNPs in the gene loci are associated with protection from the multibacillary form of the disease [22], [23].

In the present study, we hypothesized as to whether differences in the gene expression profile induced by BCG strains in comparison to *M. leprae* in a human acute monocytic leukemia cell lineage, THP-1, could be used as an experimental model to confirm previously associated genes as well as in the validation of novel susceptibility genes and pathways identified through microarrays. Also, a parallel analysis was employed where gene expression in nerve biopsies from a group of patients with leprosy or non-leprous peripheral neuropathy were tested to screen the selected gene dataset. Thus, we first used THP-1 cells infected with three different strains of BCG (Danish, Moreau and Pasteur) and, then, *M. leprae* vs Moreau using a low mycobacterial multiplicity of infection (MOI) 2:1, and established patterns of gene expression in these cells. In parallel, the same gene dataset tested in THP-1 cells were tested in a cohort of 85 nerve biopsies from leprosy and non-leprous peripheral neuropathy patients.

## Materials and Methods

### Experimental design

In Figure S1, we present a schematic representation of the design for identification of novel genes associated with leprosy immunopathogenesis.

### Human nerve biopsies

The collection of nerve biopsies was performed at ASA (from Portuguese, Ambulatório Souza Araújo, Oswaldo Cruz Institute, Fiocruz, Rio) in patients with difficult-to-diagnose nerve neuropathies [24], [25]. For diagnosis, 85 nerve biopsies were collected from patients attended at ASA. All individuals presented peripheral neuropathy and suspicion of leprosy, i.e. electroneuromyographical alterations suggestive of leprosy that needed confirmation using nerve biopsy to investigate the presence of histological architecture and *M. leprae* DNA. It is important to notice that all of these patients do not exhibit skin lesions and could not be classified according to Ridley-Jopling criteria. Clinical and laboratorial tests of serological test (anti-PGL-I), histopathological (haematoxylin & eosin and Wade staining) and PCR are performed [24], [25], [26] to support differential diagnosis of leprosy. All patients consistent with leprosy have bacilloscopic index equals to zero and were classified as pure neural leprosy according to Jardim [24] and treated as paucibacillary according to WHO recommendations. The protocol for extracting RNA and DNA from these biopsies was established for gene expression studies using nerves that had been collected for molecular and histopathological diagnosis [27], [28]. The specimens were snap-frozen in liquid nitrogen and stored at −70°C until use. Thirty-five patients (20 men and 15 women) were diagnosed with leprosy. Among those, 57% exhibited Wade positive staining in the nerve biopsy. Fifty patients (30 men and 20 women) presented other peripheral neuropathies, excluding leprosy. In this group, neurologists and pathologist were able to accurately diagnose 30 patients: entrapment neuropathy (n = 17), neurophatic vasculitis (n = 5), chronic inflammatory demyelinating polyneuropathy (n = 4); acute inflammatory demyelinating polyneuropathy (n = 1); diabetes (n = 1), HIV (n = 1), multifocal acquired demyelinating sensory and motor neuropathy (MADSAM) (n = 1). All undiagnosed patients returned to their neurological clinic for follow-up. Sample collection and procedures described in this work were approved by the Oswaldo Cruz Foundation Ethics Committee. Written informed consent was obtained from each patient. (IRB protocol/Fiocruz 151/01).

### Mycobacteria

We used three BCG strains: Danish, provided by Dr Leila Mendonça Lima (Laboratório de Genômica Funcional e Bioinformática, Instituto Oswaldo Cruz-RJ), Moreau, provided by Carolina Cavareze (Fundação Ataulpho de Paiva, RJ) and Pasteur 1173P2 WHO. They were cultured in 7H9 Middlebrook medium supplemented with 0.02% glycerol, 10% ADC Middlebrook enrichment and 0.05% Tween-80 (DIFCO Laboratories, USA), for approximately two weeks under constant agitation on a magnetic plate. Cultures were harvested in the mid-log phase, counted in a Petroff Hausser chamber according to the method described by the counter manufacturer, and kept frozen at −70°C until use.

Viable *M. leprae* Thai-53 was obtained aseptically from hind footpads of athymic nude mice [29]. Mycobacterial purity was determined by acid-fast staining and viability was determined by BacLight® Live/Dead assay (Invitrogen, USA) as described previously [30].

### Cell culture and mycobacterial infection

For microarray assays, primary human Schwann cells (SCs) from ScienCell Research Laboratories (Carlsbad, CA) were grown in Human Schwann Cell Medium (ScienCell) containing Schwann Cell Growth Serum, 5% FCS and Pen/Strep (ScienCell) and cultured for no more than 5 passages by plating in tissue culture flasks (Corning, USA) at 37°C in 5% CO_2_ until monolayers were confluent. Cells were infected with viable *M. leprae* at a multiplicity of infection (MOI) 100∶1 for 24 h at 33°C in 5% CO2 as previously described [31]. In this previous publication, SC-neuron co-cultures performed to evaluate the ability of these cells to myelinate axons even after infection (nerve regenerative response) showed that *M. leprae*–infected Schwann cells are capable of attaching to, proliferating along, and myelinating axons of cultured embryonic neurons clearly indicating a Schwann cell phenotype.

Human monocyte cell line THP-1 was obtained from the American Tissue Collection (ATCC) (Rockville, MD, USA). They were maintained in RPMI 1640 medium (LGC Biotechnology, Brazil) supplemented with 10% fetal bovine serum (FBS, HyClone Laboratories, Canada), 2 mM L-Glutamine, penicillin (100 U/mL) and streptomycin (100 U/mL). Cell cultures were kept at 37°C in a 5% CO_2_. Monocytes were seeded at 5×10^6^ cells per flask and differentiated for 24 h by incubation with 80 nM (50 ng/mL) of phorbol 12-myristate 13-acetate (PMA; Sigma-Aldrich, USA). THP-1 derived macrophages were infected with each BCG strain at a MOI 2∶1 for 24 h. Live *M. leprae* infection experiments were cultured at 33°C in 5% CO_2_. To characterize the THP-1 cell phenotype, we measured CD209, CD14, CD1b, and CD163. PMA-stimuli drive THP-1 monocytes to macrophage (CD14 positive) but not to dendritic cell (CD1b negative). A mixed population emerges whereas a higher subpopulation co-expressed CD209 and CD14, while a lower subpopulation co-expressed CD209 and CD163 (data not shown).

### RNA extraction

RNA was extracted from primary human SCs using the RNAgents-Total RNA Isolation System (Promega, Madison, WI) and from THP-1 cells and nerve biopsies using the method described by the manufacturer of Trizol reagent (Invitrogen, Life Technologies, USA). Subsequently, RNA was quantified on a Nanodrop ND-1000 spectrophotometer and integrity analyzed by agarose gel electrophoresis.

### DNA microarray assays

Microarray experiments were carried out using Human Exonic Evidence Based Oligonucleotide (HEEBO) arrays v.4.0 (Stanford Genomics Facility) containing 44,544 70-base pair oligonucleotide probes. Slides contain a) Constitutive Exonic Probes (30,718): that will recognize all known transcripts of a gene; b) Alternatively Spliced/Skipped Exonic Probes (8,441): will recognize exons that are present in some, but not all transcripts of a gene; c) Non Coding RNA Probes (196): recognizing non-protein coding transcripts (ribosomal RNAs, miRNAs); d) BCR/TCR Genic/Regional Probes (372): recognizing transcripts from genes that undergo somatic rearrangement; e) Other Probes (843): recognizing human mitochondrion derived DNA sequences; f) A total of 4,189 controls including negative, positive and doped controls. Complete details regarding the clones on the arrays may be found at: http://www.microarray.org/sfgf/heebo.do. The experimental design consisted of six competitive hybridizations between viable *M. leprae* infected (MOI 100∶1) and non-infected samples. These included three independent biological replicates, which were hybridized to duplicate slides and labeled in dye-swap. For microarray assays, 2 µg of RNA was amplified using the Message Amp II aRNA kit (Ambion, Life Technologies, USA), according to manufacturer's recommendations. Antisense RNA (aRNA) generated by this protocol was quantified and 2 µg were submitted to direct labeling of the second strand of cDNA. Labeling consisted of one round of reverse transcription by Superscript III reverse transcriptase (Invitrogen, Life Technologies) with the following modifications to the manufacturer's protocol: 2 µL random primer, 1 µL spike-in labeling control RNA (Stanford Genomics Facility), 2 µL Superscript III and a 2 hour incubation at 50°C. Subsequently, RNA was digested by adding 2 µL RNase One (Promega, Madison, WI) and 1X RNase One buffer to the sample and incubating for 10 minutes at 37°C. The remaining cDNA was purified in Microcon 30 columns (Millipore). Samples were then labeled during second strand cDNA synthesis using Bioprime DNA labeling kit (Invitrogen, Life Technologies) in a 50 µL reaction containing 120 µM of dATP, dTTP and dGTP, 60 µM non-labeled dCTP and 40 µM Alexa 555 or Alexa 647 tagged dCTP, and incubated for 3 hours at 37°C. Labeled cDNA was purified by four subsequent centrifugations at 13,000× g with 400 µL of nuclease-free water in Microcon 30 columns. Degree of labeling (DoL) was measured on a NanoDrop spectrophotometer and expressed as the percent of fluorescent nucleotides in each 100 bases. A DoL between 1 and 3% was accepted and used as quality control of fluorescent labeling. Sample pairs were joined and hybridized to HEEBO slides under coverslips in 5× SSC and 0.1% SDS by overnight incubation at 60°C in slide chambers placed in a water bath under agitation. Following hybridization, coverslips were removed and slides submitted to four washing solutions: 2× SSC and 0.2% SDS for 10 minutes, 0.5× SSC and 0.02% SDS for 10 minutes and 2× 0.1× SSC for 5 minutes each. Arrays were scanned using a GenePix 4000B microarray scanner (Molecular Devices, Sunnyvale, CA) and the hybridization signal values for the multiple probes for each mRNA were obtained with the use of GenePix Pro v.6.0 (Molecular Devices, Sunnyvale, CA). The experimental design and all microarray data have been deposited in the NCBI Gene Expression Omnibus (GEO, http://www.ncbi.nlm.nih.gov/geo accession number GSE 40950).

### DNA microarray analysis

Raw intensities were background corrected by fitting a normal-exponential convolution model to a vector of observed intensities (offset = 50). Spots that had no signal detected or a signal-to-noise ratio below or equal to 2 were filtered out and remaining data were then normalized both within arrays, using global LOWESS correction, and between arrays, using the quantile method [32]. Differentially expressed genes were ranked based on Bayesian posterior log odds. The empirical Bayes method was used to shrink the gene-wise sample variances towards common values, thus augmenting the degrees of freedom for the individual variances. This approach combines expression ratios and their variability between replicates to rank the genes [33]. Statistical significance between groups of interest was assessed for the relevant linear model contrast using moderated t-statistics. To determine which genes were significantly modulated by *M. leprae* infection, we considered differentially expressed genes according to the following comparison: SCs infected with viable *M. leprae* at a MOI 100∶1 versus SC control. We used Benjamini and Hochberg's method (1995) [34] to control the false discovery rate for the comparison. All analyses were performed using the freely available statistical software and graphics R environment [35] and the R/Bioconductor limma package [36]. Annotation of the pathways was defined using DAVID [37].

### Multiplex real-time RT-PCR

A gene set was defined based on both the microarray screening in this work as well as another work of our laboratory group (unpublished data, see File S1 for details). Other genes were included in this study after a careful review of the literature using key words such as, leprosy OR tuberculosis AND cytokines OR immune responses, SNPs, polymorphisms, genetics, although we did not use a systematic review. After revision of the literature, we selected genes associated with leprosy trying to focus on genes that 1) have been described in genetic assays, but the biological effect of the genetic association has never been assessed; 2) genes that have been tested in biological experiments (File S1). In order to test different genes we used a 96.96 chips from Fluidigm (Biomark platform) that allow us to test 96 genes in 96 different targets. Nevertheless, we used only 47 genes in duplicates for 96 samples. One slot was kept as negative control of the PCR reaction. Complementary DNA from each of the 96 samples was simultaneously pre-amplified with a mix of 47 primer pairs in a conventional thermocycler (File S1). To this end, we used Taqman preamp mastermix (Applied Biosystems, Life Technologies, USA) with 200 nM of each primer (forward and reverse) and 1.25 µl of each cDNA in a final reaction volume of 5 µl for 14 cycles. Preamplified cDNA was diluted 1∶5 in DNA suspension buffer, loaded in the Fluidigm IFC Controller HX. Each probe was placed in Taqman Gene Expression Master mix with Eva green I stain (Applied Biosystems, Life Technologies, USA) where each primer was analyzed in each sample and then amplified in the Biomark microfluidic system. Sample quality was analyzed on the company's software and raw data were exported.

### cDNA synthesis and Multiplex real-time PCR expression analysis

Complementary DNA (cDNA) produced by reverse transcription from 100 ng of total RNA using oligo (dT) primer and superscript III following manufacturer's instructions (Invitrogen, Life Technologies, USA). The fluorescence accumulation data from duplicate real-time RT-PCR reactions for each sample were used to fit four-parameter sigmoid curves to represent each amplification curve using the qpcR library [38] for the R statistical package version 2.922 [35]. The cycle of quantification, given by a characteristic point or crossing point, Cp, was determined for each amplification by the maximum of the first derivative of the fitted sigmoid curve. The efficiency of each amplification reaction was calculated as the ratio between the fluorescence of the cycle of quantification and fluorescence of the cycle immediately preceding that. The estimated efficiency of each gene was obtained by the average of all efficiencies calculated for that gene. Genes used in the normalization between the different amplified samples were selected by the methods geNorm [39] and NormFinder [40]. The comparison of means of normalized gene expression values among groups were performed either by a nonparametric one-way ANOVA with 1,000 unrestricted permutations, followed by pair-wise comparisons with Bonferroni adjustment or by a nonparametric T-test with 1,000 unrestricted permutations [41], for two or three groups respectively. Results were represented in graphs displaying the expression level mean ± standard error of mean for each group relative to the control group. Two-tailed levels of significance less than or equal to 0.01, 0.05 and 0.1 were considered as “highly significant”, “significant” and “suggestive”, respectively.

The relationship between differentially expressed gene and sample profiles was investigated by Bayesian infinite mixtures model cluster analysis [42] and represented by 2D heatmaps and dendograms. Also, a decision tree classifier [43] was trained for leprosy and non-leprous peripheral neuropathy prediction from the categorical transformation based on the cumulative distribution of the normalized expression values of the differentially expressed genes into the following linguistic variables: “very low expression”, normalized expression values below or equal to the 25^th^ quantile; “not very low”, normalized expression values below the 50^th^ quantile (median) and greater or equal to the 25^th^ quantile; “not very high”, normalized expression values below the 75^th^ quantile and greater than or equal to the 50^th^ quantile (median); “very high”, normalized expression values greater than or equal to the 75^th^ quantile. Performance of the built tree classifier was estimated by its specificity, sensitivity, and by the trapezoidal approximation of the area under the receiver operating characteristic (ROC) curve (AUC).

## Results

### Microarray screening in Schwann cells

Global gene expression analysis by microarrays was carried out to identify genes regulated in SC after 24 hours of infection by *M. leprae*. To determine which genes were significantly modulated by *M. leprae* infection, we compared SCs infected with viable *M. leprae* at a MOI 100∶1 versus control SC. After having carried out the statistical inferences, no genes were identified as differentially expressed by the selection criterion of p-value adjusted for multiple comparisons. However, by selecting genes with p-value ≤0.005, 67 genes were found to be differentially expressed, although these presented very low log fold change (LogFC) values (minimum 0.5 and maximum 1.5) (Table S1). Of these, three proteins induced by interferon type I (IFITM4, LogFC = −1.03; IFITM3 LogFC = −0.83; IFITM2, LogFC = −0.78) and eleven genes involved in oxidative phosphorylation pathway, including seven mitochondrial genes (*mt_ND1* LogFC = −1.32; *mt_ND2* LogFC = −1.12; *mt_ND2* LogFC = −0.68; *mt_ND4L* LogFC = −0.82; *mt_ND5* LogFC = −0.83; *mt_COX2* LogFC = −0,65; *mt_CYTb* LogFC = −0.63 e *mt_ATP6* LogFC = −0.62) were identified as differentially expressed.

Genes identified as differentially expressed by microarray studies were selected for functional validation by real time RT-PCR based on empirical criteria such as the observation of a larger log fold change and/or the inclusion of the gene in a pathway or enriched biological process. Thus, four mitochondrial genes were selected, mt_ND1, mt_CYTB, mt_COX2 e mt_ATP6, each of which encodes one subunit of the protein complexes involved in oxidative phosphorylation. The expression of these genes was validated by real time RT-PCR, with a result quite similar to the microarray, although not statistically significant, where infection by *M. leprae* suggests a subtle repression of mitochondrial genes in Schwann cells (Figure S2).

### Multiplex qRT-PCR

After the selection of differentially expressed genes by microarray assays (Table S1) and of others genes associated with leprosy immune pathogenesis (File S1), genes chosen for validation were analyzed using multiplex qRT-PCR. The results of these analyses are described in the next sections.

### THP-1 mRNA expression levels were similar following induction by different strains of BCG

Since different strains of BCG have been used worldwide and results suggest a variable efficacy [44], [45], [46], prior to comparison with *M. leprae* we decided to test if there are differences in the host immune response among the infection by Pasteur, Moreau or Danish BCG strains. For this, we used the THP-1 cells as a model for early innate responses upon BCG or *M. leprae* infection, since they have shown to be a good model for monocyte-derived macrophages differentiated *in vitro* and it has been used in studies of interactions between mycobacteria and human macrophages to mimic *in vivo* infection [47], [48], [49]. Transcriptional levels of 47 genes were profiled simultaneously by multiplex real-time PCR in response to BCG Danish, BCG Moreau and BCG Pasteur in THP-1 cells at 24 hours post-infection with a MOI 2∶1. Using the geNorm and NormFinder algorithms, HPRT1, RPS13a and RPL13a were determined to be a suitable set of endogenous control genes for normalization. Under the conditions tested, differences in gene transcript levels were not detected among THP-1 cells infected with any of the BCG strains used (Table S2).

### BCG Moreau and *M. leprae* induced different mRNA expression levels in THP-1

**Figure 1:**
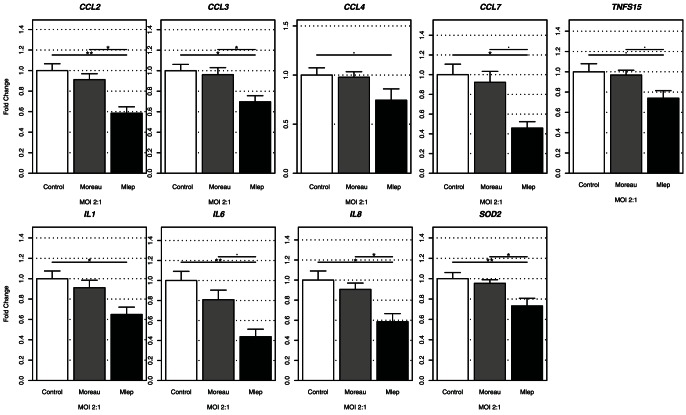
Gene expression in *M. leprae* and BCG infected THP-1 cells. THP-1 cells were infected with BCG Moreau (gray columns) or *M. leprae* (black columns) at a MOI 2∶1 for 24 hours (n = 6) or uninfected (control, white columns). Among the 47-gene set only genes that showed at least a suggestive result (n = 9) are presented. Two-tailed levels of significance less than or equal to 0.01 (**), 0.05 (*) and 0.1 (^.^) were considered as “highly significant”, “significant”, and “suggestive”, respectively.

Since there were no differences in gene expression levels induced by different BCG strains in THP-1 cells, only BCG Moreau was selected for comparison with *M. leprae* infection using a MOI 2∶1 (Table S3). We observed that mRNA expression of chemokines and cytokines, CCL2, CCL3, CCL7, IL1β, IL6 and IL8 mRNA were significantly (p<0.05) down-regulated in *M. leprae* infected THP-1 cells when compared to control (Figure 1). Most importantly, CCL2, CCL3, IL8 and SOD2 mRNA expression were down-regulated in *M. leprae* infected cells when compared to BCG Moreau infected THP-1 cells. Also, in these conditions a borderline decrease (p<0.1) was observed for CCL7, TNFS15 and IL6 (Figure 1).

### Nerves of leprosy and non-leprous patients with peripheral neuropathy displayed different gene expression profiles

**Figure 2:**
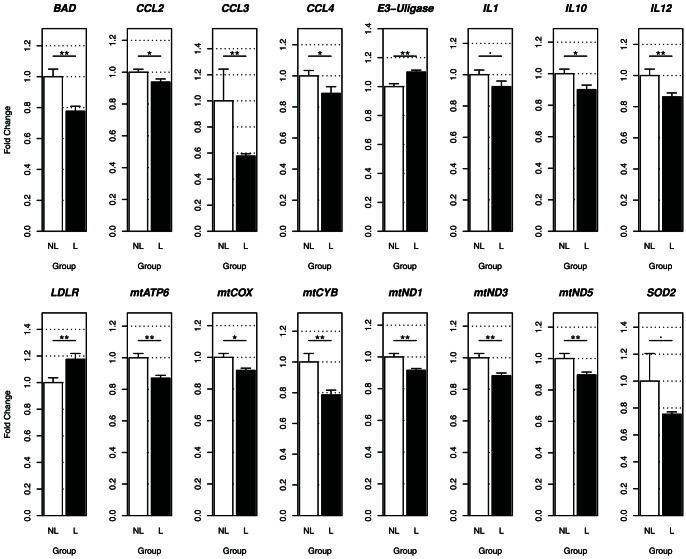
Gene expression from nerve biopsies from leprosy and non-leprous patients with peripheral neuropathy. Among the 47-gene set only genes that showed at least a suggestive result (n = 15) are presented. Two-tailed levels of significance less than or equal to 0.01 (**), 0.05 (*) and 0.1 (^.^) were considered as “highly significant”, “significant”, and “suggestive”, respectively. A total of 50 samples non-leprous patients (white columns) and 35 samples from leprosy samples (black columns).

In parallel to these results, the same set of genes was tested in nerve biopsies. RNA was extracted from a panel of samples from suspected leprosy patients. These samples are used for histopathological and molecular diagnosis. The pathological examination, along with PCR for 16S *M. leprae* DNA [27] and serological tests, that detects antibodies to the PGL-1 species-specific antigen of *M. leprae*, stratified patients as leprosy and non-leprous peripheral neuropathy. Considering Wade staining, our results indicate that the majority of leprosy patients have detectable bacilli in the histological examination (57%), while 43% had no bacilli detected in the nerve biopsies (data not shown). Gene expression levels were then compared between these groups (Table S4). Samples from leprosy patients showed down-regulation of CCL2, CCL3, CCL4 (p<0.05), while IL1β and SOD2 were borderline significant (p<0.1), confirming a repression of these genes by *M. leprae ex vivo*, as was observed *in vitro* in THP-1 (Figure 2). Other mRNAs were also less expressed in leprosy patients when compared to non-leprous samples, such as Bad, IL10, IL12 as well as several mitochondrial genes (mtCOX2, mtND1, mtND3 mtND5 mtATP6, and mtCYB). Furthermore, E3-ubiquitin ligase and *LDLR* genes were up-regulated in these leprosy patient biopsies (Figure 2).

**Figure 3:**
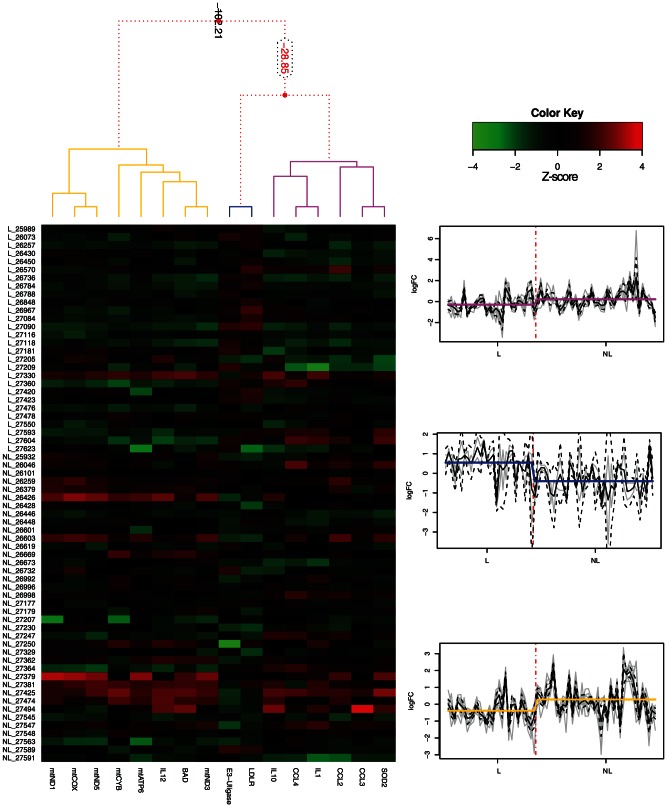
Clustering of differentially expressed genes in nerve biopsies of leprosy and non-leprous patients peripheral neuropathy. The left graph displays a dendrogram representing the 1D clusterization of genes and the 2D map corresponding to the levels of standardized gene expression profiles (z-score), while the graph on the right displays the three significant clusters (pink, blue and yellow). Red dotted lines in the dendrogram (up-left) indicate weak unions, discouraged by the Bayesian clustering analysis. Values represented in the dendrogram branches correspond to log-odds of the union of corresponding branches. Gray lines in the graphs on the right indicate gene z-scores on leprosy and non-leprous samples, while black solid and dotted lines represent the mean and CI95% of the mean for all genes belonging to each cluster, respectively. Solid pink, blue and yellow lines indicate the mean of all genes in all samples belonging to the leprosy and non-leprous groups.

**Figure 4:**
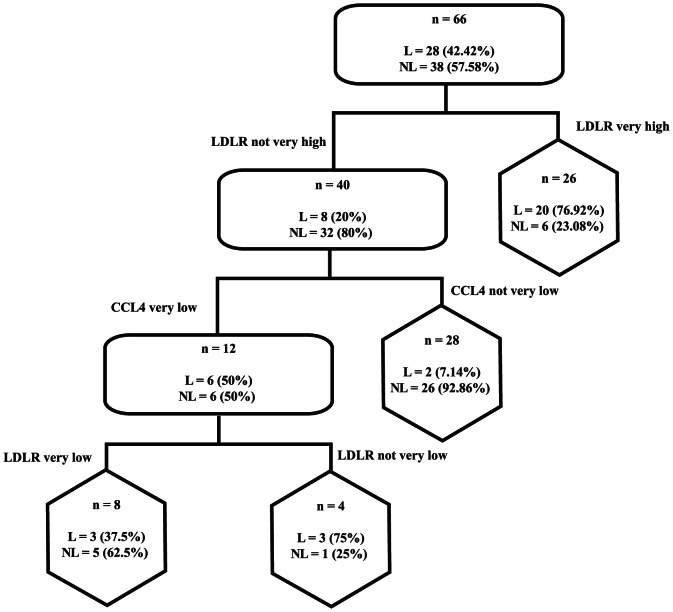
Discrimination between leprosy and non-leprous patients with peripheral neuropathy by combination of LDLR and CCL4 expression. Graph of a trained classification tree, where rounded rectangles represent internal nodes and hexagons represent terminal nodes. Percentages in each node represent the proportion of specified observations in each group.

Cluster profiles of differentially expressed genes between leprosy and non-leprous patients with peripheral neuropathy were pinpointed (Figure 3). The first cluster (pink), composed of *SOD2*, *CCL3*, *CCL2*, *IL1*, *CCL4* and *IL10*, was more repressed in leprosy patients than the second cluster (yellow), composed of mitochondrial genes, *IL12* and *Bad*, while the third cluster (blue) included only genes up-regulated in leprosy patients (E3-ligase gene and *LDLR*). Also, we determined the discriminative power of such genes after a decision tree that enables us to ascertain almost 80% of the cases as leprosy or non-leprous patients based on the mRNA expression of only 2 genes LDLR and CCL4 (Figure 4).

## Discussion

Our work has provided two very important findings: first we observed a molecular signature in leprosy patients composed of twelve differentially expressed genes, two of which were able to efficiently discriminate between leprosy and non-leprous patients. This patients have a rare leprosy condition called pure neural leprosy (PNL) and although Ridley and Jopling postulated that PNL might occur across the spectrum from LL to TT forms, in our hands the vast majority of the PNL cases are paucibacillary. Second, a clear hyporesponsive immunopathogenetic profile was observed following live *M. leprae* infection. Indeed, active induction of an immune hyporesponsive state is detected *in vitro*, which is also associated with down-modulation of *ex vivo* immune responses (anti-inflammatory and anti-apoptotic) and decreased mitochondrial metabolism. In this context, although some genes were only different in one of the situations tested (*in vitro* or *ex vivo*), results from both models reinforce the participation of specific genes and/or pathways in the pathogenesis of leprosy. *CCL2* and *CCL3*, for example, were repressed in both situations. However, other genes, such as *IL8* (in the THP-1 model) and *CCL4* (in the nerves biopsies) were down-regulated respectively, thus providing complementary information which points to the inhibition of chemokines by *M. leprae*, a possible survival strategy for the pathogen. One of the highlights of this work is that we used live *M. leprae*, which has been consistently shown to induce different pathways when compared to dead *M. leprae* in order to establish a niche for replication [50], [51]. These data also corroborate results from the multifamily genetic scan carried out in 2004, which identified the 17q21 region, enriched in chemokine genes such as *CCL2*, *CCL3*, *CCL4*, among others, as linked with leprosy. A decrease in chemokine expression could prevent activation of the host chemotactic response and, therefore, *M. leprae* could escape destruction by the immune system, contributing to the establishment of intracellular infection and consequently the spread of disease. It is important to note that these genes were selected for this study based on the genetic association study [20] that had not been validated using approaches such as those employed here.

Likewise, it is remarkable that complementarity was observed between *SOD2* mRNA repression in THP-1 and several genes associated with mitochondrial metabolism that were down-regulated in the nerves. This result also indicates that this second pathway is crucial for *M. leprae* survival, although most of the genes were only significantly reduced in nerve biopsies. Once again, it is interesting to note that *SOD2* was also suggested to participate in leprosy susceptibility through a genomic scan [14]. The role of oxidative metabolism in leprosy had not been discussed previously.

Also, it is remarkable that “classic” immune response genes such as *IL1B*, *IL6*, *IL12* and *TNF* when tested appear to confirm our models as positive controls. Previously, it has been reported that *M. leprae* induces down regulation of IL-1β, IL-6, IL-12 and TNF in monocytes, macrophages and dendritic cells [1], [2], [3]. Suppression of pro-inflammatory cytokines could be involved in the control of infection by *M. leprae* suggesting that mycobacteria may be attempting to avoid the host reaction in order to facilitate its survival. Curiously, our data also showed a borderline decrease (p<0.1) in expression of IL-6 mRNA in *M. leprae*-stimulated THP-1 cells. To this regard, another gene in the TNF superfamily, *TNFSF15*, was tested since an increased expression of this gene was observed in leprosy patient lesions [52] and genetic variants in this gene were associated to leprosy susceptibility through a genome-wide association study [6]. This association, however, was not replicated in other studies [9]. Here, we found a borderline decrease (p<0.1) of this mRNA expression in the THP-1 model and our data was unable to clarify the role of TNFSF15 in leprosy.

An increase in LDL receptor (*LDLR*) expression was observed in nerve biopsies of leprosy patients, which should lead to an increase in levels of LDL uptake. Indeed, lipid metabolism is crucial for *M. leprae* intracellular survival, and recently the accumulation of lipid derivatives in nerve lesions of LL patients was shown [51]. Also along these lines, cholesterol was described as being essential for the uptake of *Mycobacterium kansaii* and *Mycobacterium bovis* BCG by macrophages [53]. Indeed, the cholesterol content of the host's cell membrane seems to be essential for entry of mycobacteria. Therefore, the up-regulation of LDLR mRNA or protein in mycobacterial infections may increase levels of cellular cholesterol in order to facilitate entry into host cells. A classical hallmark of leprosy lesions is the presence of *M. leprae*-infected macrophages/Schwann cells showing a foamy phenotype as a result of their high lipid content [54], [55]. In the present study we showed an increase in LDL receptor mRNA expression, the main receptor of native LDL, in nerve biopsies of leprosy patients as a potential mechanism of host lipid accumulation induced by *M. leprae*. This would imply in an increase in LDL uptake leading to cholesterol accumulation in the infected tissue. Indeed, in previous studies we were able to show that cholesterol and cholesterol ester accumulate both in leprosy lesions as well as in *in vitro M. leprae* infected cells as lipid droplets [50], [51]. Moreover, we were able to show by *in vitro* assays, both in the context of Schwann cells and macrophages, that *M. leprae* infection is able to increase host cell uptake of native LDL (unpublished data). Cholesterol metabolism has shown to play an important role in mycobacterial pathogenesis both by facilitating bacterial internalization [51] as well as by acting as a carbon and energy source [56], [57]. Therefore, the up-regulation of LDLR mRNA in *M. leprae* infected nerves may increase levels of cellular cholesterol in order to facilitate entry and bacterial survival into host cells. In fact, it also raises an interesting possibility whereas detection of lipid metabolites could help diagnosis. A recent study performed on serum samples from patients before and after MDT conclusion (Amaral et al., unpublished data) confirmed previous observations by Al Mubarak and coworkers [58] showing that polyunsaturated fatty acid (PUFA) metabolism is deeply affected during leprosy.

Another gene that was down regulated in leprosy patient samples was *Bad*, which is a pro-apoptotic gene. *Bad* was also suppressed after infection of THP-1 by *M. leprae* in a previous study [59] where authors observed that irradiated *M. leprae* inhibits apoptosis by diminishing pro-apoptotic Bad and Bak mRNA expression, while inducing the anti-apoptotic gene Mcl-1. These results could be combined with the induction of E3-ubiquitin ligases that also elicit an anti-apoptotic response. Indeed, *PARK2*, which is another E3-ubiquitin ligase, was previously shown to be associated with leprosy [14]. Also, it is quite interesting that two different E3-ubiquitin ligases were associated with leprosy using different approaches (genomic scan and microarray) suggesting that indeed ubiquitination is a crucial process, which possibly regulates apoptosis and is involved in leprosy susceptibility. Once more, we observe a coherent pattern of gene expression where *M. leprae* induces an anti-apoptotic response in the host cell, which would be an appropriate strategy for this intracellular pathogen that only multiplies every 14 days. It has been shown that Parkin is involved in the ubiquitination of Bcl-2, which prevents cytochrome c release from the mitochondria [60] preventing apoptosis. In fact, the regulation of mitochondrial functions, as observed in the results of this work, may also channel the cell in the same direction. We showed a decrease in mitochondrial gene expression in Schwann cells after infection with *M. leprae*. The very same genes were also down-regulated in nerve biopsies from leprosy patients suggesting a suppression of mitochondrial energy metabolism. Furthermore, SOD2, a mitochondrial gene involved in defense against superoxide (O2-) and other toxic reactive oxygen species (ROS) was also down-regulated in THP-1 cells infected with *M. leprae*. The mitochondrial alterations observed are consistent with inhibition of apoptosis, which are often triggered in the mitochondria. These organelles play a central role as a stress sensor within the cell and the repression of mitochondrial genes could be a confirmation of the hypothesis of an anti-apoptotic effect caused by *M. leprae*. The mitochondrial alterations have already been associated with other disease models such as Chagas' disease, in which down-regulation of several transcripts encoding components of the mitochondrial oxidative phosphorylation pathway was observed in a murine model infected by *Trypanosoma cruzi* [61], suggesting a decrease in global energy production and subsequent cardiac performance.

In this context, when we selected differentially expressed genes to cluster between leprosy and non-leprous patients, a distinctive pattern was found to distinguish between both groups. Significantly regulated genes clustered in three unique sets of genes, one of which was almost entirely composed of mitochondrial genes involved in the electron transport chain and found to be down-regulated in leprosy patients. Nonetheless, a classification tree was developed, based on the mRNA expression of only two genes, *LDLR* and *CCL4*, each of which belonged to a cluster with an opposite pattern of regulation in leprosy patients, either up or down, respectively. CCL4 was also observed recently as a potential biomarker to distinguish leprosy patients and controls of areas endemic, as Brazil [62]. The discriminative power of these two genes was able to ascertain almost 80% of the cases as leprosy or non-leprous and this is extremely important in patients with difficult diagnosis, as the case of pure neural leprosy, which the pathology is restricted to the nerve and the patient did not presents skin lesions [24].

Furthermore, this study is the first to show an induction of genes induced by type-I IFN by *M. leprae* in cells of the peripheral nervous system: IFITM2, IFITM3, IFITM4. The type-I IFN-inducible genes had recently been observed to be more induced in skin lesions of leprosy patients with disseminated lepromatous form compared to patients with tuberculoid form [63]. This result is consistent with other recent reports of global gene expression which identified this class of genes as induced in blood of patients with active tuberculosis compared to patients with latent tuberculosis [64], [65], [66] and identified a highly prominent type-I interferon molecular signature in three models of TB (newly-diagnosed TB patients followed-up through treatment, a BCG infected human macrophage cell line and an *in vivo* mouse lung TB infection model) [67]. An interferon-related signature in THP-1 cells infected by different *M.tb* strains was also identified [68]. In addition, another study in our laboratory found other members of this family, to be up-regulated in primary SCs infected by live *M. leprae* (unpublished data), consistent with the microarray analysis present here. The role of type I IFN in mycobacterial diseases still needs to be further evaluated.

In summary, our findings suggest that decrease of various genes related to immune response and mitochondrial metabolisms against infection by *M. leprae* indicating that *M. leprae* appears to have a suppressive effect in human cells may be associated with negative regulation of the immune response and therefore for success of the infection.

## Supporting Information

Figure S1 **Schematic representation of the experimental design for identification of novel genes associated with immunopathogenesis of leprosy.**
(DOCX)

Figure S2 **Normalized gene expression values of the chosen genes by conventional qRT-PCR from the list of DE genes in microarray experiments.**
(DOC)

File S1 **List of genes used and the primer sequences in multiplex real-time PCR.**
(DOC)

Table S1 **Differentially expressed genes (p-value ≤0.005) in control Schwann Cells versus** ***M. leprae*** **-infected Schwann cells for 24 hours.**
(DOC)

Table S2 **Normalized values of gene expression in THP-1 cells either uninfected or infected with BCG Danish, BCG Moreau or BCG Pasteur strains at a MOI 2∶1 for 24 hours (n = 10).**
(DOC)

Table S3 **Normalized values of gene expression in THP-1 cells either uninfected or infected with BCG Moreau or** ***M. leprae*** **at a MOI 2∶1 for 24 hours (n = 6).**
(DOC)

Table S4 **Normalized gene expression values of nerve biopsy samples from leprosy (n = 35) and non-leprous peripheral neuropathy patients (n = 50).**
(DOC)
