# Supplementary material for: Gene Expression Profiling Specifies Chemokine, Mitochondrial and Lipid Metabolism Signatures in Leprosy
Source: PLoS One. 2013 Jun 14;8(6):e64748. doi: 10.1371/journal.pone.0064748 (PMC3683049; doi:10.1371/journal.pone.0064748)

Figure S1 – Schematic representation of the experimental design for identification of novel genes associated with immunopathogenesis of leprosy.


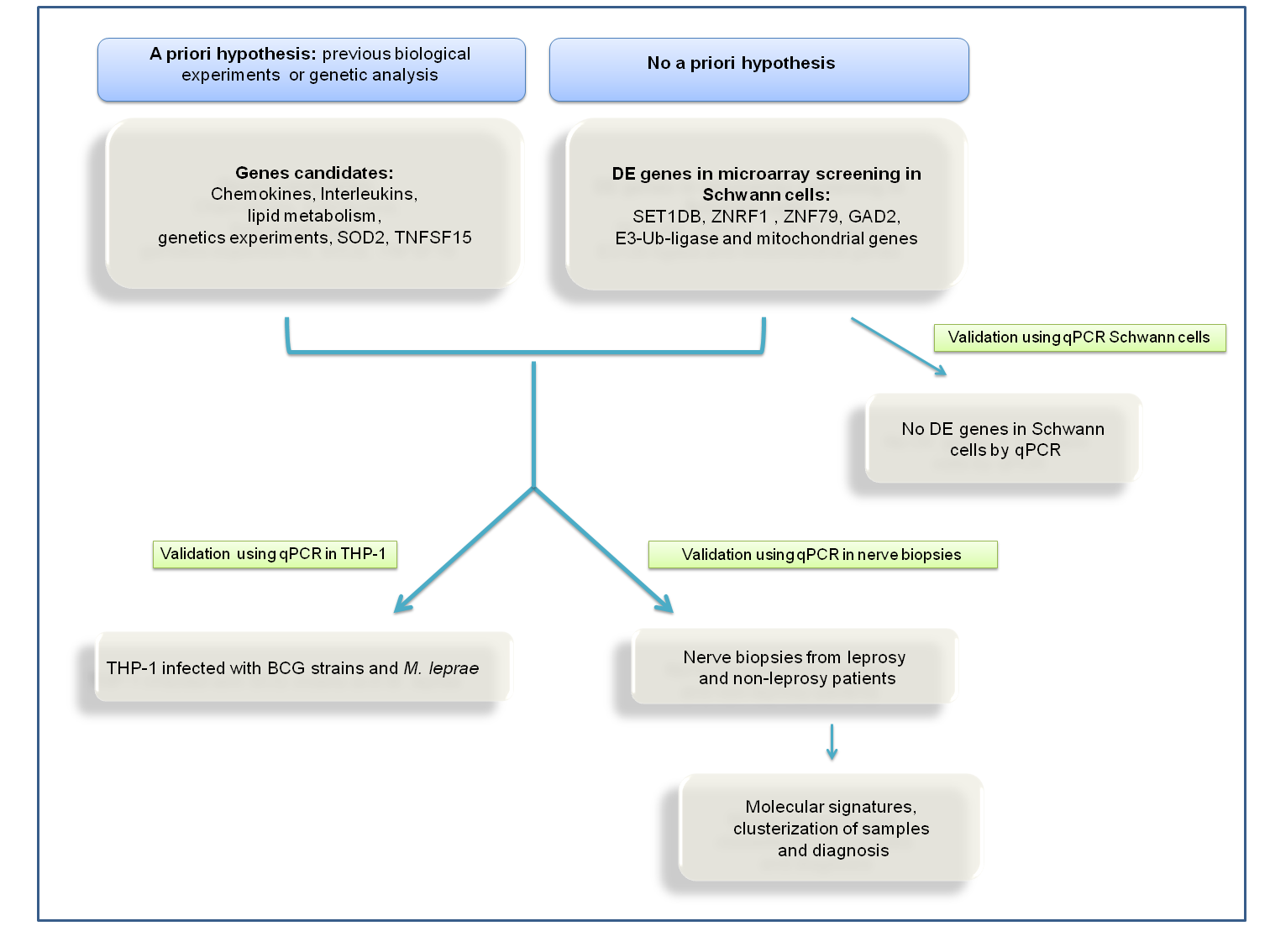

Supplement: Figure S1 — Schematic representation of the experimental design for identification of novel genes associated with immunopathogenesis of leprosy. (DOCX) [file pone.0064748.s001.docx]
