# Supplementary material for: Gene Expression Profiling Specifies Chemokine, Mitochondrial and Lipid Metabolism Signatures in Leprosy
Source: PLoS One. 2013 Jun 14;8(6):e64748. doi: 10.1371/journal.pone.0064748 (PMC3683049; doi:10.1371/journal.pone.0064748)

Figure S2 - Normalized gene expression values ​​of the chosen genes by conventional qRT-PCR from the list of DE genes in microarray experiments.


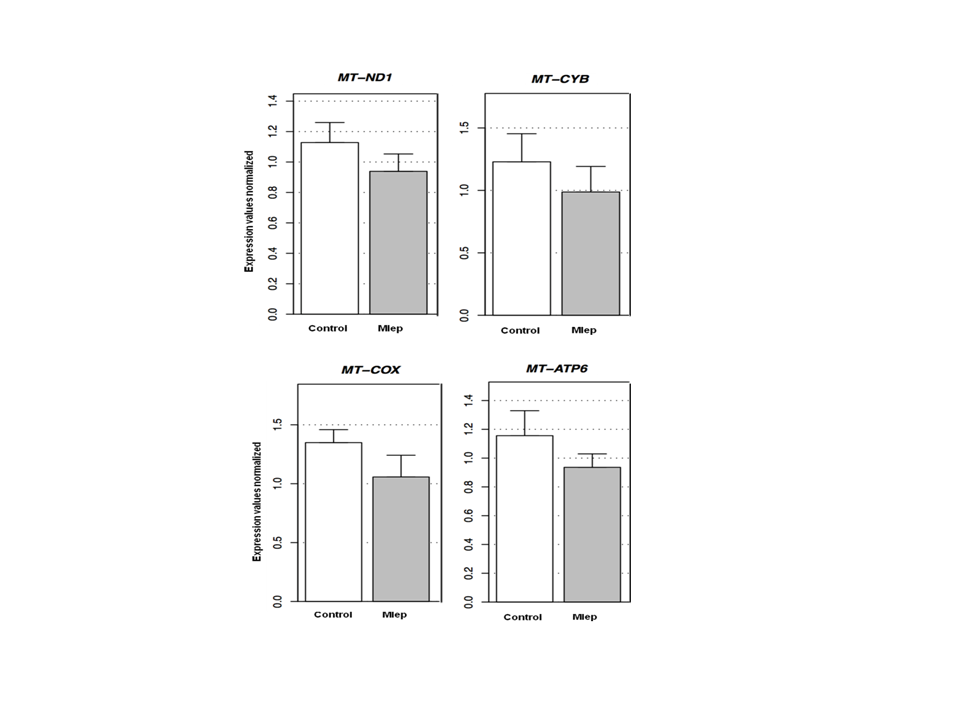

Supplement: Figure S2 — Normalized gene expression values of the chosen genes by conventional qRT-PCR from the list of DE genes in microarray experiments. (DOC) [file pone.0064748.s002.doc]
