# Supplementary material for: Gene Expression Profiling Specifies Chemokine, Mitochondrial and Lipid Metabolism Signatures in Leprosy
Source: PLoS One. 2013 Jun 14;8(6):e64748. doi: 10.1371/journal.pone.0064748 (PMC3683049; doi:10.1371/journal.pone.0064748)
Supplement: File S1 — List of genes used and the primer sequences in multiplex real-time PCR. (DOC) [file pone.0064748.s003.doc]

File S1 – List of genes and primer sequences used in multiplex real-time PCR.

| **Number** | **Gene** | **Strand** | **Sequence (5' - 3')** | **Gene selection strategy** | **Reference** |
| --- | --- | --- | --- | --- | --- |
| 1 | GAPDH | Forward | AGTGATGGCATGGACTGTGGTCAT | C^a^ | - |
|  |  | Reverse | CAACAGCCTCAAGATCATCAGCAA |  |  |
| 2 | HPRT1 | Forward | GTGGAAGATATAATTGACACTGGC | C | - |
|  |  | Reverse | AACACTTCGTGGGGTCCTTT |  |  |
| 3 | RPL13a | Forward | GACAAGAAAAAGCGGATGGT | C | - |
|  |  | Reverse | GTACTTCCAGCCAACCTCGT |  |  |
| 4 | RPS13a | Forward | CGAAAGCATCTTGAGAGGAACA | C | - |
|  |  | Reverse | TCGAGCCAAACGGTGAATC |  |  |
| 5 | CCL2 | Forward | CCTGCTGTTATAACTTCACCAAT | B^b^ | [20, 69^c^] |
|  |  | Reverse | TTGAAGATCACAGCTTCTTTGG |  |  |
| 6 | CCL3 | Forward | TGCTGCTTCAGCTACACCTC | B, G^d^ | [20, 70] |
|  |  | Reverse | GCTTCGCTTGGTTAGGAAGA |  |  |
| 7 | CCL4 | Forward | CGCCTGCTGCTTTTCTTACA | B, G | [20, 70] |
|  |  | Reverse | TGCTTCTTTTGGTTTGGAAT |  |  |
| 8 | CCL5 | Forward | CCTGCTGCTTTGCCTACATT | B, G | [20, 70] |
|  |  | Reverse | GGGTGACAAAGACGACTGCT |  |  |
| 9 | CCL7 | Forward | CTTCAACTACCTGCTGCTACAGA | B, G | [20, 71] |
|  |  | Reverse | CAGTTTGGTCTTGAAGATTACAGC |  |  |
| 10 | TNF | Forward | GAGGGTTTGCTACAACATGG | B, G | [18] |
|  |  | Reverse | CCCCAGGGACCTCTCTCTA |  |  |
| 11 | IL-6 | Forward | GCATCTAGATTCTTTGCCTTTTT | B, G | [72] |
|  |  | Reverse | GAAAATCATCACTGGTCTTTTGG |  |  |
| 12 | IL-10 | Forward | ATCGATGACAGCGCCGTAG | B, G | [19] |
|  |  | Reverse | GATGCCCCAAGCTGAGAAC |  |  |
| 13 | IL-12 | Forward | TTTCTTTTCTCTCTTGCTCTTGC | B | [73] |
|  |  | Reverse | GTGGAGGTCAGCTGGGAGTA |  |  |
|  |  |  |  |  |  |

|  | **Gene** | **Strand** | **Sequence (5' - 3')** | **Gene selection strategy** | **Reference** |
| --- | --- | --- | --- | --- | --- |
| 14 | IL-1 | Forward | CAAAATACCTGTGGCCTTGG | B | [74] |
|  |  | Reverse | TTTTTGGGATCTACACTCTCCAG |  |  |
| 15 | IL-8 | Forward | CTGCGCCAACACAGAAATTA | B | [70] |
|  |  | Reverse | TGAATTCTCAGCCCTCTTCAA |  |  |
| 16 | NOX3 | Forward | CTGGGACGAAAATACTGACG | G | [14] |
|  |  | Reverse | AATACTGCTGCTGGGGTGAT |  |  |
| 17 | SOD2 | Forward | GGAACGGGGACACTTACAAA | G | [14] |
|  |  | Reverse | TAAGCGTGCTCCCACACAT |  |  |
| 18 | NOD2 | Forward | CACTGATGCTGGCAAAGAAC | G | [6] |
|  |  | Reverse | GGTAGGTGATGCAGTTATTGGA |  |  |
| 19 | SET1DB | Forward | AGCTCAAGTTCCGGAAGAAA | M^e^ | unpublished data |
|  |  | Reverse | GCGATCACCTGACGGATATT |  |  |
| 20 | mtND1 | Forward | TCCGCTACGACCAACTCATA | M | This study |
|  |  | Reverse | GGGGAATGCTGGAGATTGTA |  |  |
| 21 | mtND2 | Forward | ACTAGCCCCCATCTCAATCA | M | This study |
|  |  | Reverse | ATTTTGCGTAGCTGGGTTTG |  |  |
| 22 | mtND3 | Forward | GCGTCCCTTTCTCCATAAAA | M | This study |
|  |  | Reverse | GGCTCATGGTAGGGGTAAAA |  |  |
| 23 | mtND4L | Forward | TCACACCTCATATCCTCCCTACTAT | M | This study |
|  |  | Reverse | GGAGTGGGTGTTGAGGGTTAT |  |  |
| 24 | mtND5 | Forward | AGGCGCTATCACCACTCTGT | M | This study |
|  |  | Reverse | TTGGTTGATGCCGATTGTAA |  |  |
| 25 | mtCOX2 | Forward | GGCCTGACTGGCATTGTATT | M | This study |
|  |  | Reverse | TCAGTGAATGAAGCCTCCTATG |  |  |
| 26 | mtCYTb | Forward | AGACGCCCTCGGCTTACTT | M | This study |
|  |  | Reverse | ATGTGGGGAGGGGTGTTTA |  |  |
|  |  |  |  |  |  |
| **Number** | **Gene** | **Strand** | **Sequence (5' - 3')** | **Gene selection strategy** | **Reference** |
| 27 | mtATP6 | Forward | GCCACCTACTCATGCACCTAAT | M | This study |
|  |  | Reverse | TTAAGGCGACAGCGATTTCTA |  |  |
| 28 | Ninjurin | Forward | CTTGACAAGGAAGATGAGCAG | B, G | [75, 76] |
|  |  | Reverse | AAGGCCGTCGTGGAACAG |  |  |
| 29 | BCL2 | Forward | CCCCTGGTGGACAACATC | B | [46] |
|  |  | Reverse | CAGCCAGGAGAAATCAAACAG |  |  |
| 30 | IDO1 | Forward | GGGAAGACCCAAAGGAGTTT | B | [77] |
|  |  | Reverse | GGAGGAACTGAGCAGCATGT |  |  |
| 31 | IDO2 | Forward | TGCTTCATGCCTTTGATGAG | B | [77] |
|  |  | Reverse | GGAAGGTGCTGAGTGGATGT |  |  |
| 32 | LRRK2 | Forward | TGATGACAGCACAGCTAGGAA | G | [6] |
|  |  | Reverse | CCAAACGGTCAAGCAAGATT |  |  |
| 33 | PINK1 | Forward | GCCTCCAGACGTGAGACAG | G | [6] |
|  |  | Reverse | CACCCCAGAGGCTTAGATGA |  |  |
| 34 | ZNRF1 | Forward | TGCTTGCTGACTCCTCTCAA | M | unpublished data |
|  |  | Reverse | GGAGGAAGGGAGAACCATGA |  |  |
| 35 | TNFSF15 | Forward | CAGGAGTTTGCACCTTCACA | G | [6] |
|  |  | Reverse | TGCTGTGTGGGAGTTTGTCT |  |  |
| 36 | RIPK2 | Forward | GGGATAGCACCATTTCTGGA | G | [6] |
|  |  | Reverse | ATACCAGGCTGCAGACGTTC |  |  |
| 37 | ZNF79 | Forward | TCCAGGAGCCTTGTCCTACT | G | unpublished data |
|  |  | Reverse | CTCTGGTGGTGATCCAGATATAA |  |  |
| 38 | C6orf136 | Forward | CTTGCTCTTTTTGCGGTTCT | G | [6] |
|  |  | Reverse | TAGACGATGGCGACAAATGA |  |  |
| **Number** | **Gene** | **Strand** | **Sequence (5' - 3')** | **Gene selection strategy** | **Reference** |
| 39 | E3-Ub-ligase | Forward | CCATCTTCACTGAGCAGGAGTTA | M | unpublished data |
|  |  | Reverse | GAACCACTGCATCTGAATAGAGTTG |  |  |
| 40 | BAK | Forward | ACGACATCAACCGACGCTAT | B | [46] |
|  |  | Reverse | CACTCTCAAACAGGCTGGTG |  |  |
| 41 | BAD | Forward | GAGGACGACGAAGGGATG | B | [46] |
|  |  | Reverse | TCCCTTCTTAAAGGAGTCCACA |  |  |
| 42 | MIF | Forward | CGCAGAACCGCTCCTACA | B | [78] |
|  |  | Reverse | GAGTTGTTCCAGCCCACATT |  |  |
| 43 | GAD2 | Forward | CGGCAACTCACCAAGACATT | B | unpublished data |
|  |  | Reverse | AAGTTTCTCAGTAGGGGGCTTAG |  |  |
| 44 | LTA4H | Forward | AATTGAATGAGTTTTTAGCACAGA | G | [22] |
|  |  | Reverse | GCAGCCATCTGAATCGTATTT |  |  |
| 45 | PPARgama | Forward | AGTCCTCACAGCTGTTTGCAAGC | B | [79] |
|  |  | Reverse | GAGCGGGTGAAGACTCATGTCTGT |  |  |
| 46 | LPL | Forward | AAAGGCACCTGCGGTATTT | B | [50] |
|  |  | Reverse | TCACATGCCGTTCTTTGTTC |  |  |
| 47 | LDLR | Forward | ACCAGGACGGCTACAGCTAC | B | [80] |
|  |  | Reverse | GAGGTCTCAGGAAGGGTTCTG |  |  |

^a^ Constitutive (C) genes were used for the normalization of the assays;

^b^ Biological (B)- genes that were selected after revision of the literature for biological studies;

^c^all references that were not cited previously in the text are listed here (numbers 69-80, see below);

^d^ Genetic (G): genes were selected after revision of the literature for genetic studies;

^e^ Microarray (M): two different data sets (two independent microarrays experiments) were used. The data set presented as “this study” can be found at GSE 40950 and are described in the results section. The other data set was defined as “unpublished results” (GSE35423) and are being compiled for another paper. The references cited are from works based to choose genes involved directly or indirectly with leprosy.

Supplementary References:

69. Kirkaldy AA, Musonda AC, Khanolkhar-Young S, Suneetha S, Lockwood DN (2003) Expression of CC and CXC chemokines and chemokine receptors in human leprosy skin lesions. Clin Exp Immunol 134: 447-453.

70. Méndez-Samperio P and Vázpuez A (2003) Infection of human monocytes with *Mycobacterium bovis* BCG induces production of CC-chemokines. J Infect 47: 139–147.

71. Ragno S, Romano M, Howell S, Pappin D, Jenner P, et al. (2001) Changes in gene expression in macrophages infected with *Mycobacterium tuberculosis*: a combined transcriptomic and proteomic approach. Immunology 104 :99-108.

72. Sousa AL, Fava VM, Sampaio LH, Martelli CM, Costa MB, et al. (2012) Genetic and immunological evidence implicates interleukin 6 as a susceptibility gene for leprosy type 2 reaction. J Infect Dis 205: 1417-1424.

73. Libraty DH, Airan LE, Uyemura K, Jullien D, Spellberg B, et al. (1997) Interferon-gamma differentially regulates interleukin-12 and interleukin-10 production in leprosy. J Clin Invest 99: 336-341.

74. Yamamura M, Uyemura K, Deans RJ, Weinberg K, Rea TH, et al. (1991) Defining protective responses to pathogens: cytokine profiles in leprosy lesions. Science 254: 277-279.

75. Cardoso CC, Martinez AN, Guimarães PE, Mendes CT, Pacheco AG, et al. (2007) Ninjurin 1 asp110ala single nucleotide polymorphism is associated with protection in leprosy nerve damage. J Neuroimmunol 190: 131-138.

76. Graça CR, Paschoal VD, Cordeiro-Soubhia RM, Tonelli-Nardi SM, Machado RL, et al. (2012) NINJURIN1 single nucleotide polymorphism and nerve damage in leprosy. Infect Genet Evol 12: 597-600.

77. de Souza-Sales J, Lara FA, Amadeu TP, de Oliveira Fulco T, da Costa Nery JA, et al. (2011) The role of indoleamine 2, 3-dioxygenase in lepromatous leprosy immunosuppression. Clin Exp Immunol 165: 251-263.

78. Yamada G, Shijubo N, Takagi-Takahashi Y, Nishihira J, Mizue Y, et al. (2002) Elevated levels of serum macrophage migration inhibitory factor in patients with pulmonary tuberculosis. Clin Immunol 104: 123-127.

79. Almeida PE, Silva AR, Maya-Monteiro CM, Töröcsik D, D'Avila, et al. (2009) Mycobacterium bovis bacillus Calmette-Guérin infection induces TLR2-dependent peroxisome proliferator-activated receptor gamma expression and activation: functions in inflammation, lipid metabolism, and pathogenesis. J Immunol 183: 1337-1345.

80. Gatfield J and Pieters J (2000) Essential role for cholesterol in entry of mycobacteria in macrophages. Science 288: 1647-1650.
