# Supplementary material for: Gene Expression Profiling Specifies Chemokine, Mitochondrial and Lipid Metabolism Signatures in Leprosy
Source: PLoS One. 2013 Jun 14;8(6):e64748. doi: 10.1371/journal.pone.0064748 (PMC3683049; doi:10.1371/journal.pone.0064748)
Supplement: Table S1 — Differentially expressed genes (p-value ≤0.005) in control Schwann Cells versus M. leprae -infected Schwann cells for 24 hours. (DOC) [file pone.0064748.s004.doc]

Table S2 - Differentially expressed genes (p-value ≤ 0.005) in control Schwann Cells versus *M. leprae*-infected Schwann cells for 24 hours.

| **Gene** | **Description** | **log fold change** |
| --- | --- | --- |
| LOC391269 | similar to ankyrin repeat domain 20 family, member A2 | -1,466 |
| MTND1 | NADH dehydrogenase subunit 1 | -1,320 |
| CFL1 | cofilin 1 (non-muscle) | -1,225 |
| MTND2 | NADH dehydrogenase subunit 2 | -1,120 |
| IFITM4P | 6p22.1, interferon induced pseudogene | -1,034 |
| LOC283412 | similar to 60S ribosomal protein L29 | -0,984 |
| GANAB | glucosidase, alpha; neutral AB | -0,898 |
| C6orf48 | chromosome 6 open reading frame 48 | -0,880 |
| MT1JP | metallothionein 1J (pseudogene) | -0,862 |
| PRKCSH | protein kinase C substrate 80K-H | -0,844 |
| EFEMP2 | EGF-containing fibulin-like extracellular matrix protein 2 | 0,842 |
| MTND5 | NADH dehydrogenase subunit 5 | -0,830 |
| PLP2 | proteolipid protein 2 (colonic epithelium-enriched) | -0,830 |
| CST3 | cystatin C (amyloid angiopathy and cerebral hemorrhage) | -0,825 |
| LOC144383 | Interferon-induced transmembrane protein 3 | -0,825 |
| MTND4 | NADH dehydrogenase subunit 4 | -0,823 |
| RPS25 | ribosomal protein S25 | -0,807 |
| RPL21 | ribosomal protein L21 | -0,805 |
| IFITM2 | interferon induced transmembrane protein 2 (1-8D) | -0,789 |
| RPS24P15 | similar to ribosomal protein S24 | -0,775 |
| UQCRQ | ubiquinol-cytochrome c reductase, complex III subunit VII, 9.5kDa | -0,772 |
| mtRNA_RNR1 | mitochondrially encoded 12S ribosomal RNA | -0,764 |
| MTND4L | NADH dehydrogenase subunit 4L | -0,761 |
| LOC389308 | similar to ribosomal protein L10a | -0,742 |
| NRM | nurim (nuclear envelope membrane protein) | -0,726 |
| LOC389127 | similar to 40S ribosomal protein S10 | -0,713 |
| DBI | diazepam binding inhibitor (GABA receptor modulator) | -0,707 |
| RPL12P6 | ribosomal protein L12 pseudogene 6 | -0,707 |
| LOC146053 | similar to ribosomal protein S3a | -0,701 |
| COMMD7 | COMM domain containing 7 | 0,698 |
| PKM2 | pyruvate kinase, muscle | -0,697 |
| RPS26P6 | ribosomal protein S26 pseudogene 6 | -0,691 |
| LOC285900 | similar to 60S ribosomal protein L6 | -0,689 |
| LOC441377 | similar to 40S ribosomal protein S26 | -0,689 |
| PRDX2 | peroxiredoxin 2 | -0,686 |
| mtRNA_ND3 | mitochondrially encoded NADH dehydrogenase subunit 3 | -0,685 |
| GSTP1 | glutathione S-transferase pi | -0,665 |
| mtRNA_COX-2 | cytochrome c oxidase subunit II | -0,648 |
| HIST1H4C | 6p21.3 histone 1, H4c | -0,647 |
| LOC402287 | ribosomal protein S3a pseudogene | -0,644 |

Suppl. table 2 cont.

| **Gene** | **Description** | **log fold change** |
| --- | --- | --- |
|  |  |  |
| PPIAL3 | peptidylprolyl isomerase A (cyclophilin A)-like 3 | -0,641 |
| CHMP4A | chromatin modifying protein 4ª | -0,630 |
| SERPINE1 | serpin peptidase inhibitor, clade E | 0,630 |
| NDUFB10 | NADH dehydrogenase (ubiquinone) 1 beta subcomplex, 10, 22kDa | -0,628 |
| SLC25A28 | solute carrier family 25, member 28 | 0,627 |
| mtRNA_CYTB | mitochondrially encoded cytochrome b | -0,627 |
| OXA1L | oxidase (cytochrome c) assembly 1-like | 0,619 |
| LOC388460 | hypothetical LOC388460 | -0,618 |
| SLC16A3 | solute carrier family 16 (monocarboxylic acid transporters), member 3 | 0,614 |
| TPI1 | triosephosphate isomerase 1 | -0,598 |
| LOC441777 | similar to Serine/threonine-protein kinase (PLK-1) (STPK13) | 0,587 |
| LOC440733 | similar to 40S ribosomal protein S15 (RIG protein) | -0,580 |
| HSPC148 | hypothetical protein HSPC148 | -0,578 |
| C10orf70 | chromosome 10 open reading frame 70 | -0,574 |
| CACNG7 | calcium channel, voltage-dependent, gamma subunit 7 | 0,573 |
| KRTHA1 | keratin, hair, acidic, 1 | -0,570 |
| LOC402175 | hypothetical gene | -0,570 |
| RPS26e | ribosomal protein S26-like | -0,564 |
| RPS26P10 | ribosomal protein S26 pseudogene 10 | -0,562 |
| RPS13 | ribosomal protein S13 | -0,556 |
| RPS3A | ribosomal protein S3A | -0,552 |
| SPOCD1 | SPOC domain containing 1 | 0,549 |
| PTD008 | PTD008 protein | -0,549 |
| PIN4 | NIMA-interacting protein, 4 (parvulin) | -0,537 |
| ECHS1 | enoyl Coenzyme A hydratase, short chain, 1, mitochondrial | -0,529 |
| SMAP | small acidic protein | 0,515 |
| NR0B2 | nuclear receptor subfamily 0, group B, member 2 | 0,509 |

Pink, genes induced by IFN type I. Grey, genes involved in oxidative phosphorylation pathway. Annotation taken from DAVID [36].
