# Supplementary material for: Gene Expression Profiling Specifies Chemokine, Mitochondrial and Lipid Metabolism Signatures in Leprosy
Source: PLoS One. 2013 Jun 14;8(6):e64748. doi: 10.1371/journal.pone.0064748 (PMC3683049; doi:10.1371/journal.pone.0064748)
Supplement: Table S2 — Normalized values of gene expression in THP-1 cells either uninfected or infected with BCG Danish, BCG Moreau or BCG Pasteur strains at a MOI 2∶1 for 24 hours (n = 10). (DOC) [file pone.0064748.s005.doc]

Table S3 - Normalized values of gene expression in THP-1 cells either uninfected or infected with BCG Danish, BCG Moreau or BCG Pasteur strains at a MOI 2:1 for 24 hours (n=10).

|  | **Global** | **Control vs. BCG Danish** | | **Control vs. BCG Moreau** | | **Control vs. BCG Pasteur** | | **BCG Danish vs. BCG Moreau** | | | **BCG Danish vs. BCG Pasteur** | | **BCG Moreau vs. BCG Pasteur** | |
| --- | --- | --- | --- | --- | --- | --- | --- | --- | --- | --- | --- | --- | --- | --- |
|  | **p.value** | **logFC** | **p.value** | **logFC** | **p.value** | **logFC** | **p.value** | **logFC** | | **p.value** | **logFC** | **p.value** | **logFC** | **p.value** |
| **BAD** | 1 | 0,024 | 1 | 0,012 | 1 | 0,005 | 1 | -0,012 | 1 | | -0,019 | 1 | -0,007 | 1 |
| **BAK** | 0,79 | 0,195 | 1 | -0,054 | 1 | 0,182 | 1 | -0,249 | 1 | | -0,013 | 1 | 0,236 | 1 |
| **BCL2** | 0,757 | -0,014 | 1 | 0,012 | 1 | 0,071 | 1 | 0,025 | 1 | | 0,084 | 1 | 0,059 | 1 |
| **C6orf136** | 0,919 | 0,038 | 1 | 0,009 | 1 | -0,021 | 1 | -0,029 | 1 | | -0,060 | 1 | -0,031 | 1 |
| **CCL2** | 0,802 | 0,097 | 1 | 0,128 | 1 | -0,007 | 1 | 0,030 | 1 | | -0,105 | 1 | -0,135 | 1 |
| **CCL3** | 0,673 | 0,104 | 1 | 0,058 | 1 | -0,056 | 1 | -0,046 | 1 | | -0,159 | 0,831 | -0,114 | 1 |
| **CCL4** | 0,738 | 0,057 | 1 | 0,013 | 1 | -0,110 | 1 | -0,044 | 1 | | -0,168 | 0,971 | -0,123 | 1 |
| **CCL5** | 0,844 | 0,024 | 1 | -0,061 | 1 | -0,048 | 1 | -0,085 | 1 | | -0,072 | 1 | 0,013 | 1 |
| **CCL7** | 0,746 | -0,134 | 1 | 0,133 | 1 | -0,132 | 1 | 0,267 | 1 | | 0,002 | 1 | -0,265 | 1 |
| **E3-Uligase** | 0,549 | 0,133 | 1 | 0,010 | 1 | -0,050 | 1 | -0,123 | 1 | | -0,183 | 0,651 | -0,059 | 1 |
| **IDO1** | 0,259 | -0,090 | 1 | 0,098 | 1 | -0,171 | 1 | 0,189 | 0,855 | | -0,081 | 1 | -0,269 | 0,196 |
| **IDO2** | 0,702 | 0,042 | 1 | -0,051 | 1 | -0,090 | 1 | -0,093 | 1 | | -0,132 | 1 | -0,039 | 1 |
| **IL1** | 0,648 | 0,228 | 0,839 | 0,131 | 1 | 0,051 | 1 | -0,097 | 1 | | -0,178 | 1 | -0,081 | 1 |
| **IL10** | 0,843 | -0,038 | 1 | -0,070 | 1 | 0,051 | 1 | -0,032 | 1 | | 0,089 | 1 | 0,121 | 1 |
| **IL12** | 0,911 | -0,074 | 1 | 0,018 | 1 | 0,010 | 1 | 0,092 | 1 | | 0,085 | 1 | -0,008 | 1 |
| **IL6** | 0,542 | 0,096 | 1 | 0,349 | 0,679 | 0,171 | 1 | 0,253 | 1 | | 0,075 | 1 | -0,178 | 1 |
| **IL8** | 0,632 | 0,206 | 1 | 0,107 | 1 | -0,035 | 1 | -0,099 | 1 | | -0,241 | 0,835 | -0,142 | 1 |
| **LDLR** | 0,769 | -0,002 | 1 | -0,207 | 1 | -0,099 | 1 | -0,205 | 1 | | -0,097 | 1 | 0,107 | 1 |
| **LPL** | 0,958 | 0,056 | 1 | 0,039 | 1 | 0,002 | 1 | -0,018 | 1 | | -0,054 | 1 | -0,037 | 1 |
| **LRRK2** | 0,94 | -0,039 | 1 | -0,008 | 1 | 0,001 | 1 | 0,031 | 1 | | 0,039 | 1 | 0,009 | 1 |
| **LTA4H** | 0,927 | -0,026 | 1 | 0,005 | 1 | -0,006 | 1 | 0,031 | 1 | | 0,020 | 1 | -0,011 | 1 |
| **MIF** | 0,852 | 0,055 | 1 | -0,026 | 1 | -0,004 | 1 | -0,080 | 1 | | -0,059 | 1 | 0,021 | 1 |
| **mtATP6** | 0,81 | -0,076 | 1 | 0,006 | 1 | -0,009 | 1 | 0,082 | 1 | | 0,067 | 1 | -0,015 | 1 |
| **mtCOX** | 0,785 | -0,034 | 1 | 0,004 | 1 | 0,071 | 1 | 0,038 | 1 | | 0,105 | 1 | 0,067 | 1 |
| **mtCYB** | 0,988 | -0,024 | 1 | 0,013 | 1 | 0,042 | 1 | 0,038 | 1 | | 0,066 | 1 | 0,028 | 1 |
| **mtND1** | 0,723 | -0,101 | 1 | 0,018 | 1 | -0,027 | 1 | 0,119 | 1 | | 0,074 | 1 | -0,045 | 1 |
| **mtND2** | 0,795 | -0,068 | 1 | 0,000 | 1 | -0,012 | 1 | 0,068 | 1 | | 0,056 | 1 | -0,012 | 1 |
| **mtND3** | 0,894 | -0,073 | 1 | -0,006 | 1 | 0,024 | 1 | 0,067 | 1 | | 0,097 | 1 | 0,030 | 1 |
| **mtND4L** | 0,679 | -0,027 | 1 | -0,036 | 1 | 0,050 | 1 | -0,009 | 1 | | 0,077 | 1 | 0,086 | 1 |
| **mtND5** | 0,889 | -0,072 | 1 | 0,018 | 1 | -0,023 | 1 | 0,089 | 1 | | 0,049 | 1 | -0,040 | 1 |
| **NINJURIN** | 0,964 | 0,025 | 1 | -0,030 | 1 | 0,045 | 1 | -0,055 | 1 | | 0,020 | 1 | 0,075 | 1 |
| **NOD2** | 0,943 | 0,010 | 1 | 0,037 | 1 | 0,079 | 1 | 0,027 | 1 | | 0,069 | 1 | 0,042 | 1 |
| **PINK1** | 0,965 | 0,049 | 1 | 0,022 | 1 | 0,018 | 1 | -0,027 | 1 | | -0,031 | 1 | -0,004 | 1 |
| **PPARg** | 0,875 | -0,045 | 1 | 0,021 | 1 | -0,008 | 1 | 0,066 | 1 | | 0,037 | 1 | -0,029 | 1 |
| **RIPK2** | 0,244 | 0,038 | 1 | 0,087 | 0,951 | -0,052 | 1 | 0,049 | 1 | | -0,090 | 0,807 | -0,139 | 0,188 |
| **SET1DB** | 0,934 | -0,045 | 1 | -0,160 | 1 | -0,156 | 1 | -0,115 | 1 | | -0,111 | 1 | 0,004 | 1 |
| **SOD2** | 0,695 | 0,050 | 1 | 0,054 | 1 | -0,054 | 1 | 0,004 | 1 | | -0,104 | 1 | -0,108 | 1 |
| **TNF** | 0,822 | 0,030 | 1 | 0,143 | 1 | 0,063 | 1 | 0,113 | 1 | | 0,033 | 1 | -0,080 | 1 |
| **TNFS15** | 0,53 | 0,250 | 0,691 | 0,023 | 1 | 0,144 | 1 | -0,227 | 0,939 | | -0,106 | 1 | 0,121 | 1 |
| **ZNF79** | 0,532 | 0,112 | 0,763 | 0,008 | 1 | 0,056 | 1 | -0,105 | 0,799 | | -0,057 | 1 | 0,048 | 1 |
| **ZNRF1** | 0,863 | 0,041 | 1 | 0,008 | 1 | -0,026 | 1 | -0,033 | 1 | | -0,068 | 1 | -0,035 | 1 |
