# Supplementary material for: Gene Expression Profiling Specifies Chemokine, Mitochondrial and Lipid Metabolism Signatures in Leprosy
Source: PLoS One. 2013 Jun 14;8(6):e64748. doi: 10.1371/journal.pone.0064748 (PMC3683049; doi:10.1371/journal.pone.0064748)
Supplement: Table S3 — Normalized values of gene expression in THP-1 cells either uninfected or infected with BCG Moreau or M. leprae at a MOI 2∶1 for 24 hours (n = 6). (DOC) [file pone.0064748.s006.doc]

Table S4 - Normalized values of gene expression in THP-1 cells either uninfected or infected with BCG Moreau or *M. leprae* at a MOI 2:1 for 24 hours (n=6).

|  | **Global** | **Control vs. BCG Moreau** | | **Control vs. M. leprae** | | **BCG Moreau vs. M. leprae** | |
| --- | --- | --- | --- | --- | --- | --- | --- |
|  | **p.value** | **logFC** | **p.value** | **logFC** | **p.value** | **logFC** | **p.value** |
| **BAD** | 0,521 | 0,012 | 1 | 0,209 | 0,875 | 0,196 | 0,941 |
| **BAK** | 0,799 | -0,054 | 1 | -0,219 | 1 | -0,165 | 1 |
| **BCL2** | 0,945 | 0,012 | 1 | -0,018 | 1 | -0,029 | 1 |
| **C6orf136** | 0,992 | 0,009 | 1 | -0,004 | 1 | -0,013 | 1 |
| **CCL2** | 0,003 | 0,128 | 1 | 0,771 | 0,003 | 0,643 | 0,018 |
| **CCL3** | 0,020 | 0,058 | 1 | 0,514 | 0,021 | 0,456 | 0,036 |
| **CCL4** | 0,064 | 0,013 | 1 | 0,465 | 0,081 | 0,452 | 0,129 |
| **CCL5** | 0,221 | -0,061 | 1 | 0,100 | 0,863 | 0,161 | 0,219 |
| **CCL7** | 0,024 | 0,133 | 1 | 1,080 | 0,030 | 0,947 | 0,054 |
| **E3-Uligase** | 0,541 | 0,010 | 1 | -0,142 | 0,863 | -0,152 | 0,899 |
| **IDO1** | 0,423 | 0,098 | 1 | 0,327 | 0,381 | 0,228 | 0,938 |
| **IDO2** | 0,906 | -0,051 | 1 | -0,054 | 1 | -0,002 | 1 |
| **IL1** | 0,042 | 0,131 | 1 | 0,617 | 0,039 | 0,486 | 0,123 |
| **IL10** | 0,902 | -0,070 | 1 | -0,047 | 1 | 0,024 | 1 |
| **IL12** | 0,360 | 0,018 | 1 | 0,243 | 0,536 | 0,225 | 0,614 |
| **IL6** | 0,004 | 0,349 | 0,833 | 1,230 | 0,003 | 0,881 | 0,063 |
| **IL8** | 0,012 | 0,107 | 1 | 0,767 | 0,021 | 0,660 | 0,045 |
| **LDLR** | 0,361 | -0,207 | 0,875 | -0,309 | 0,572 | -0,103 | 1 |
| **LPL** | 0,880 | 0,039 | 1 | -0,053 | 1 | -0,092 | 1 |
| **LRRK2** | 0,972 | -0,008 | 1 | -0,017 | 1 | -0,009 | 1 |
| **LTA4H** | 0,601 | 0,005 | 1 | 0,063 | 0,965 | 0,057 | 1 |
| **MIF** | 0,667 | -0,026 | 1 | 0,080 | 1 | 0,106 | 1 |
| **mtATP6** | 0,903 | 0,006 | 1 | 0,048 | 1 | 0,042 | 1 |
| **mtCOX** | 0,265 | 0,004 | 1 | 0,181 | 0,381 | 0,177 | 0,423 |
| **mtCYB** | 0,462 | 0,013 | 1 | 0,222 | 0,710 | 0,209 | 0,779 |
| **mtND1** | 0,780 | 0,018 | 1 | 0,090 | 1 | 0,071 | 1 |
| **mtND2** | 0,755 | 0,000 | 1 | 0,066 | 1 | 0,066 | 1 |
| **mtND3** | 0,661 | -0,006 | 1 | 0,127 | 1 | 0,133 | 1 |
| **mtND4L** | 0,798 | -0,036 | 1 | 0,015 | 1 | 0,051 | 1 |
| **mtND5** | 0,443 | 0,018 | 1 | 0,174 | 0,554 | 0,157 | 0,833 |
| **NINJURIN** | 0,767 | -0,030 | 1 | -0,119 | 1 | -0,089 | 1 |
| **NOD2** | 0,200 | 0,037 | 1 | 0,269 | 0,252 | 0,231 | 0,405 |
| **PINK1** | 0,955 | 0,022 | 1 | 0,037 | 1 | 0,015 | 1 |
| **PPARg** | 0,461 | 0,021 | 1 | -0,106 | 0,968 | -0,127 | 0,617 |
| **RIPK2** | 0,162 | 0,087 | 0,641 | 0,152 | 0,189 | 0,065 | 1 |
| **SET1DB** | 0,871 | -0,160 | 1 | -0,180 | 1 | -0,021 | 1 |
| **SOD2** | 0,005 | 0,054 | 1 | 0,461 | 0,003 | 0,407 | 0,027 |
| **TNF** | 0,187 | 0,143 | 1 | 0,360 | 0,195 | 0,217 | 0,818 |
| **TNFS15** | 0,032 | 0,023 | 1 | 0,428 | 0,066 | 0,405 | 0,066 |
| **ZNF79** | 0,991 | 0,008 | 1 | 0,010 | 1 | 0,003 | 1 |
| **ZNRF1** | 0,849 | 0,008 | 1 | 0,048 | 1 | 0,039 | 1 |
