# Supplementary material for: Gene Expression Profiling Specifies Chemokine, Mitochondrial and Lipid Metabolism Signatures in Leprosy
Source: PLoS One. 2013 Jun 14;8(6):e64748. doi: 10.1371/journal.pone.0064748 (PMC3683049; doi:10.1371/journal.pone.0064748)
Supplement: Table S4 — Normalized gene expression values of nerve biopsy samples from leprosy (n = 35) and non-leprous peripheral neuropathy patients (n = 50). (DOC) [file pone.0064748.s007.doc]

Table S5 - Normalized gene expression values of nerve biopsy samples from leprosy (n=35) and non-leprous peripheral neuropathy patients (n=50).

| **Gene** | **L vs. NL** | |
| --- | --- | --- |
|  | **logFC** | **p.value** |
| **BAD** | -0,336276549 | <0,001 |
| **BAK** | 0,081247762 | 0,27 |
| **BCL2** | -0,066089872 | 0,27 |
| **C6orf136** | -0,016939223 | 0,6 |
| **CCL2** | -0,090138467 | 0,03 |
| **CCL3** | -0,368504137 | 0,01 |
| **CCL4** | -0,185868806 | 0,02 |
| **CCL5** | -0,157800143 | 0,39 |
| **CCL7** | -0,185959309 | 0,17 |
| **E3-Uligase** | 0,149079861 | <0,001 |
| **IDO1** | -0,1100742 | 0,29 |
| **IL1** | -0,118377398 | 0,1 |
| **IL10** | -0,15153553 | 0,02 |
| **IL12** | -0,188933368 | 0,01 |
| **IL6** | -0,028290842 | 0,6 |
| **LDLR** | 0,231571285 | <0,001 |
| **LPL** | -0,09641678 | 0,53 |
| **LRRK2** | -0,080524056 | 0,85 |
| **LTA4H** | -0,015961969 | 0,84 |
| **MIF** | -0,129574073 | 0,44 |
| **mtATP6** | -0,188377156 | <0,001 |
| **mtCOX** | -0,113347887 | 0,01 |
| **mtCYB** | -0,311940593 | <0,001 |
| **mtND1** | -0,117189887 | 0,01 |
| **mtND2** | -0,044288155 | 0,33 |
| **mtND3** | -0,16201121 | <0,001 |
| **mtND4L** | -0,047444982 | 0,32 |
| **mtND5** | -0,140177995 | 0,01 |
| **NINJURIN** | 0,030981739 | 0,47 |
| **NOD2** | -0,140513371 | 0,73 |
| **PINK1** | -0,098113112 | 0,68 |
| **PPARg** | -0,161874493 | 0,36 |
| **RIPK2** | 0,020293816 | 0,72 |
| **SET1DB** | -0,274797396 | 0,23 |
| **SOD2** | -0,16530016 | 0,1 |
| **TNF** | -0,106160967 | 0,91 |
| **TNFS15** | -0,001217082 | 0,99 |
| **ZNF79** | -0,101406341 | 0,91 |
| **ZNRF1** | -0,122720098 | 0,5 |
